# Two multi-temporal datasets to track debris flow after the 2008 Wenchuan earthquake

**DOI:** 10.1038/s41597-022-01658-y

**Published:** 2022-08-27

**Authors:** Lei Wang, Ming Chang, Jian Le, Lanlan Xiang, Zhang Ni

**Affiliations:** 1 State Key Laboratory of Geohazard Prevention and Geoenvironment Protection, Chengdu University of Technology, Chengdu, 610059 China; 2 Sichuan Xingshu Engineering Survey And Design Group CO., LTD, Chengdu, 610072 China

**Keywords:** Natural hazards, Hydrology

## Abstract

We provide two datasets for tracking the debris flow induced by the 2008 Wenchuan Mw 7.9 earthquake on a section of the Longmen mountains on the eastern side of the Tibetan plateau (Sichuan, China). The database was obtained through a literature review and field survey reports in the epicenter area, combined with high-resolution remote sensing image and extensive data collection and processing. The first dataset covers an area of 892 km^2^, including debris flows from 2008 to 2020 (an updated version). 186 debris flows affecting 79 watersheds were identified. 89 rainfall stations were collected to determine the rainfall events for the post-earthquake debris flow outbreak. The second database is a list of mitigation measures for post-earthquake debris flows, including catchment name, check dam number, coordinates, construction time, and successful mitigation date. The datasets can aid different applications, including the early warning and engineering prevention of post-earthquake debris flow, as well as provide valuable data support for research in related disciplines.

## Abstract (summary)

**Table:** 

Measurement(s)	debris flow • water-based rainfall • check dam
Technology Type(s)	satellite imaging • weather station
Sample Characteristic - Organism	earthquake induced debris flows and mitigation measures
Sample Characteristic - Environment	earthquake
Sample Characteristic - Location	Wenchuan County

## Background & Summary

Large, continental earthquakes can cause considerable disruptions in erosion and sediment export patterns from mountain ranges^1–9^. Strong earthquakes create numerous co-seismic landslides, degrade the terrain and deposit much debris on slopes and channels^10–14^. As time passes, these materials will be pushed into low-order channel and eventually deposited^15–19^. Under heavy rainfall, these sediments will turn into debris flows^20,21^.

Debris flow is a type of special torrent containing numerous solid materials, and it usually erupts suddenly and has extremely destructive. The activity of debris flows increased significantly after the earthquake, which brought major threats to human lives and infrastructure^22^, such as the 1923 Kanto earthquake in Japan^23^, the 1999 Chi-Chi earthquake in Taiwan^4,24,25^, the 2005 Kashmir earthquake in Pakistan^26^, the 2008 earthquake in Wenchuan^27–31^, the 2010 earthquake in Haiti^32^, the 2013 earthquake in Lushan^33^, and the 2017 earthquake in Jiuzhaigou, China^34^.

To mitigate the impact and threat of debris flows after earthquakes, many researchers have carried out extensive research on the early warning and mitigation measures of debris flow^35–40^. High-efficiency early warning methods such as real-time catchment monitoring^35,41^ (rain gauge, mud level meters, ultrasonic flow meters, real-time video cameras) and statistical analysis-based debris flow initiation rainfall threshold are widely used in large-area debris flow prevention. Some studies have found that the rainfall threshold decreased significantly after the earthquake and then gradually recovered^4,42,43^. A rainfall event has multiple rainfall parameters, which affect the research results differently. Under the action of soil infiltration, precipitation increases the pore water pressure and reduces the effective stress, which affects the slope stability^22^. In addition, some studies suggest that the rainfall pattern has an important influence on the debris flow initiation^13,44^.

Recovering from large earthquakes is a challenge, especially in mountainous areas, where post-earthquake disaster risks can significantly impact over a long period^29,45^. For the prevention and mitigation of debris flows, engineering measures are usually constructed in the earthquake-affected regions^46^. Check dams, flexible barriers, silt dams, and baffle arrays are commonly used for debris flow control^47^. These mitigation measures can reduce the energy of debris flows, control surface erosion that occurs in upstream areas, and play an important role in geologic hazard prevention. Some studies have shown that the dam’s location in the channel is essential for effectively slowing and controlling debris flows^39,48,49^. Choi, *et al*.^39^ investigated the effect of barrier locations, especially source-to-barrier distance, on debris flows velocity and volume using smoothed particle hydrodynamics (SPH). Dai, *et al*.^49^ analyzed the impact force of debris flow on the check dam after the Wenchuan earthquake by numerical simulation method. However, the monitoring and control measures of debris flow require a lot of time and effort because few data and records of post-earthquake debris flows can be freely available^41^.

**Fig. 1:**
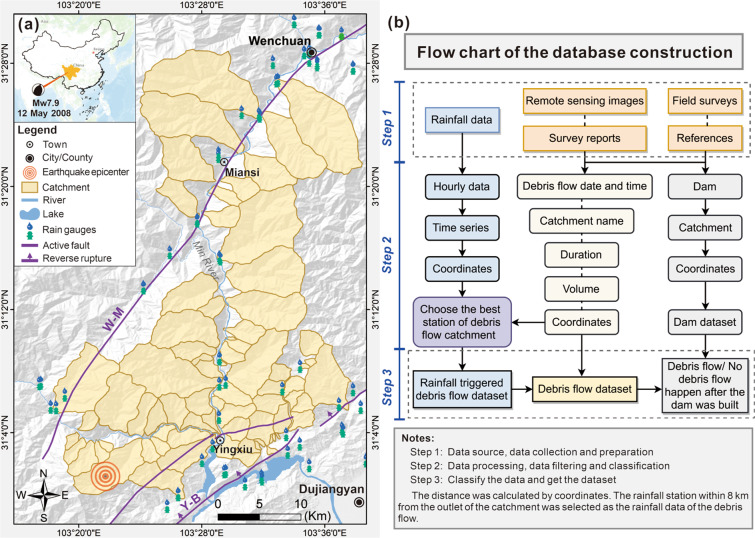
General view of the study area and the flow chart in Wenchuan Country, Sichuan, China; (**a**) details of the area in which the multi-temporal dataset was carried out, details of the area in which debris flows were recorded, with the indication of rain gauges and check dam; (**b**) flow chart of the database construction.

The debris flow in Wenchuan area was active before the 2008 earthquake^50^. Many debris flows occurred in the Longmen Mountains area after the 2008 Wenchuan earthquake^13^ (Fig. 1a). By the end of 2010, more than 440 debris flows happened in the earthquake-stricken area^6,51^. Such as the “9.24” catastrophic debris flow event in 2008, the “August 13” event in 2010, the “July 10” catastrophe event in 2013^30,52–54^, and the “August 20” debris flow event in 2019^55–57^. Following the Wenchuan earthquake, many researchers are working on the mechanism, prediction, and early warning of post-earthquake debris flow^6,27,30,31,35,41,43,51,56–59^. How to prevent and control the post-earthquake debris flow has become a prominent and urgent research topic^22^. Targeted reconstruction work has been carried out in the earthquake area, effectively preventing debris flow in some areas and ensuring the safety of residents’ lives and property^60^. According to the field investigation results and literature, most of these catchments remain natural without disposal. Successful prevention and control cases are important to learn from and summarize^46^. These debris flow events and mitigation measures provide valuable data for studying post-earthquake debris flow early warning and mitigation measures^61^.

However, existing public data has many time gaps that need to be filled^41,62^, and there is still a lack of accessible public data on debris flows after the Wenchuan earthquake and no multi-temporal disaster mitigation measures datasets that are freely available. In this study, we focus on the watershed from Yingxiu Town to Wenchuan County, along the bank of the Min River after the 2008 Wenchuan earthquake. Two datasets were supplied that track debris flows events. Through data collection, interpretation of high-resolution remote sensing images, and field investigation, 186 debris flows were identified in the first dataset that affected 79 catchments. The total area of these catchments is approximately 892 km^2^ (Fig. 1). 89 rainfall stations were collected, covering an epicenter area of 1566 km^2^. The second dataset contains a list of debris flow mitigation measures from 2008 to 2020, including the catchment name, dam number, construction period, and coordinates. The flow chart of the database construction is shown in Fig. 1b. Our datasets are freely available at 10.5281/zenodo.6891244^63^. We also encourage other scholars to share their relevant data, which can help improve the current dataset to facilitate post-earthquake debris flow research.

## Methods

The study area is along the Minjiang River from Yingxiu Town to Wenchuan County and the Longchi and Yuzixi river basins, including 79 watersheds with an area of 892 km^2^ (Fig. 1). Combining literature review, field investigation reports, and remote sensing images, we constructed two datasets of debris flow events and mitigation measures after the 2008 Wenchuan earthquake. The first dataset covers an area of 892 km^2^, including debris flows from 2008 to 2020. 186 debris flows affecting 79 watersheds were identified. 89 rainfall stations were collected to determine the rainfall events for the post-earthquake debris flow outbreak. The second database is a list of mitigation measures for post-earthquake debris flows, including catchment name, check dam number, coordinates, construction time, and successful mitigation date. The detailed database build flow chart is shown in Fig. 1b.

**Fig. 2:**
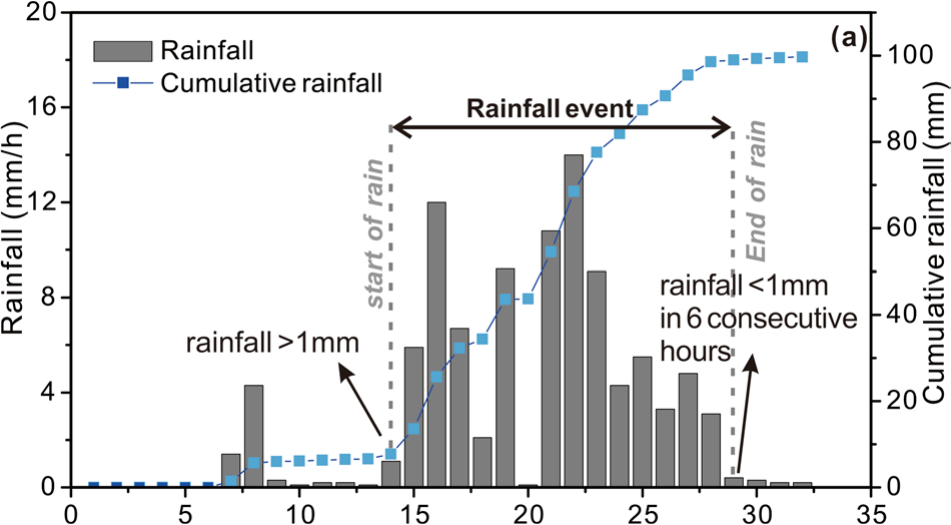
The classification standard of rainfall events used in the study. (The classification standard of rainfall events in this study by Zhou *et al*., 2013).

According to the recorded debris flow events, we went to the field to investigate and interview residents every year after the rainstorm. GPS and laser range finders were used to measure the coordinates, and debris flows fan thickness. According to the field survey reports, the volume of the material washed out by the debris flow is calculated. The hourly rainfall data was from The Meteorological Administration of China and the Meteorological Bureau of Sichuan Province, recorded by an automatic rain gauge. The data obtained from the rainfall station are usually time-continuous series, which cannot be directly used for analysis. Therefore, it is necessary to pre-process the data to extract the rainfall events. In this study, a standard proposed by Zhou and Tang^64^ was used to divide the rain events. This standard regards the hourly rainfall of >1 mm as the beginning of rainfall and <1 mm for 6 consecutive hours as the end of the rainfall^64^ (Fig. 2).

As for the triggering rainfall of each debris flow event, the coordinates of rain gauges were used to calculate the closest distance to the debris flow event catchment. To provide more data for researchers and consider the distribution density of rainfall data we collected, the rain gauges within 8 km (the mean mainstream length) of the debris flow were selected^41^. Most debris flow events in the first dataset were collected from literature review and survey reports (see the file named “data references” in the debris flow repository). For the bigger and most catastrophic events, we conducted field investigations and interviews with the residents. Remote sensing imagery validated debris flow events (Table 1).

**Table 1:** List of remote sensing images used in this study.

ID	Acquisition date	Image source	Resolution
1	2008/5/23	Airbus photos /RGB-panchromatic /Google earth	0.51 m
2	2011/4/26	Airbus photos /RGB-panchromatic/Google earth	0.51 m
3	2014/12/7	Airbus photos /RGB-panchromatic/Google earth	0.51 m
4	2015/4/15	Airbus photos /RGB-panchromatic/Google earth	0.51 m
5	2018/4/3	Image 2021 CNES/Airbus photos/RGB-panchromatic/Google earth	1.02 m
6	2019/10/29	Image 2021 CNES/Airbus photos/RGB-panchromatic/Google earth	1.02 m
7	2021/11/14	Image 2021 CNES/Airbus photos/RGB-panchromatic /Google earth	0.51 m

## Data Records

Our dataset is available at 10.5281/zenodo.6891244^63^.

### Dataset of multi-temporal debris flow events after the wenchuan earthquake

**Fig. 3:**
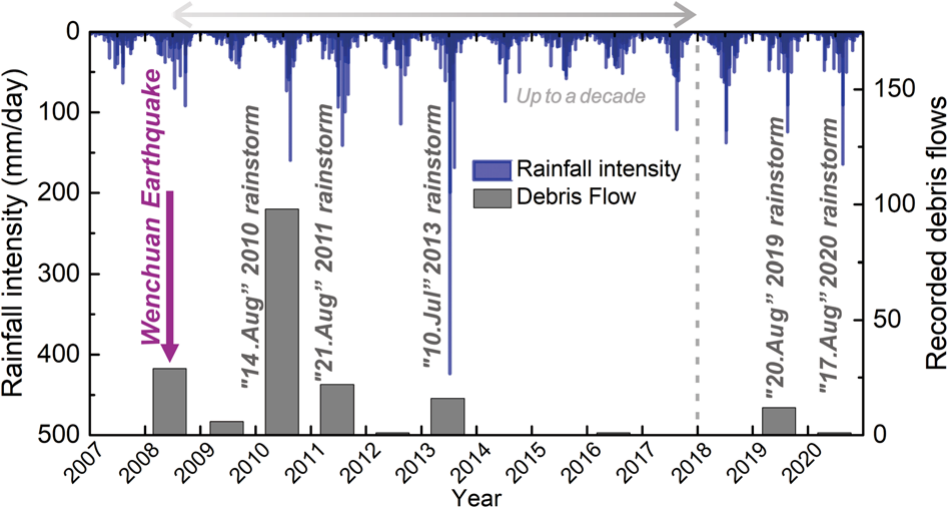
The catastrophic debris flows in the study area and the corresponding daily rainfall.

The first dataset is about the recorded debris flow events after the 2008 Wenchuan earthquake. Figure 3 shows the detailed number of these debris flow events from 2008–2020. The structure of the dataset is summarised in Table 2. The first dataset contains information about recorded debris flows (DF_DATA) events and their triggering rainfalls (DF_R_DATA). The debris flow (DF_DATA) events are stored in the “.xlsx” file format in the dataset. It includes information such as DF_ID, gully name, coordinates, date and time, deposition volumes, and data references. Rain gauge data includes the rain gauge ID (RG_ID), coordinates, altitude, amount of rain, temporal resolution, units, and other information. The debris flow triggering rainfall data (DF_R_DATA) are stored in “.txt” file format and includes DF_ID, RG_ID, debris flow date and time, rainfall, and units. Not only the catastrophic debris flows from 2008–2020^7,27,31,43,53,56,57^, but also some small events are included in this dataset. Some small debris flows usually lack records in the previous literature because of their long distance, or do not cause serious damage to the downstream residential areas or buildings.

**Table 2:** Structure of the dataset of debris flows and their triggering rainfalls.

Filename	File type	Layers (sheets)	Attributes (columns)
DF_DATA	Spreadsheet (.csv)	Debris flows	DF_ID, gully name, coordinates, date and time, deposition volumes, and data references.
	Polygon (.shp)	Catchments	DF_ID, C_ID, catchment name, catchment area.
DF_R_DATA	Folder file (.txt)	DF_R	RG_ID, DF_ID, debris flow date and time, rainfall, units

Attributes: DF_ID: identifier of the debris flows; C_ID: identifier of the catchment to which the debris flows belong; RG_ID: identifier of the rain gauge; Each folder contains the rain gauges located within a distance of 8 km for a given event.

### Triggering rainfalls of multi-temporal debris flows

**Fig. 4:**
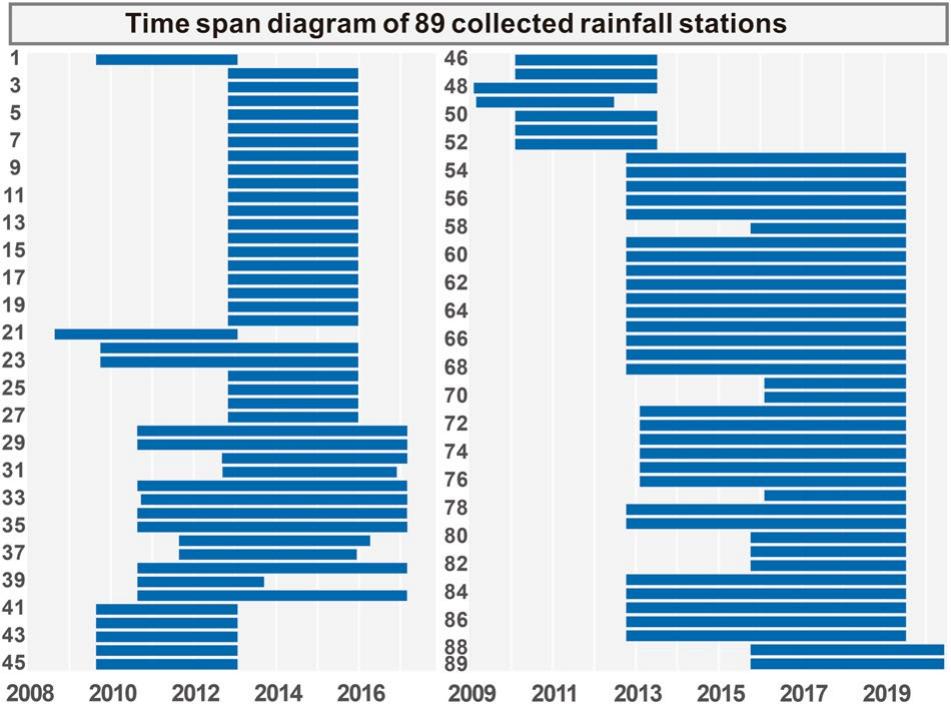
Time span of collected rainfall data in the database from 2008 to 2020.

Our first dataset contains 89 rainfall stations from Wenchuan country to Yingxiu Town, covering an area of 1566 km^2^, including the catchments along the Minjiang River, Longchi, and Yuzixi River, with the period from 2008 to 2020 (Fig. 4). Table 2 shows the structure of the first dataset.

**Fig. 5:**
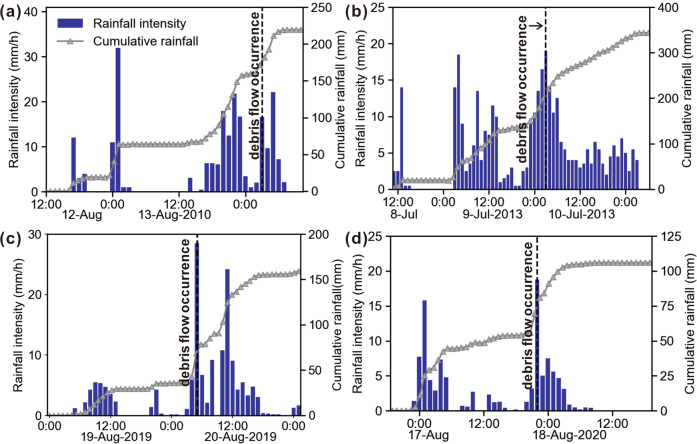
Catastrophic debris flow and rainstorm in the study area. (**a**) Hourly and accumulated precipitation in the Hongchun catchment on August 14, 2010 (C_ID W25). A rainfall station in the Yingxiu catchment recorded rainfall data; (**b**) hourly and accumulated precipitation in the Er catchment on July 10, 2013 (C_ID W7). Rainfall data was recorded by a rainfall gauge located in Er. (**c**) Hourly and accumulated precipitation in the Cutou catchment (C_ID W70), caused catastrophic debris flow on August 20, 2019. (**d**) Hourly and accumulated precipitation in the Taoguan catchment (C_ID W48).

The name of the saved folder corresponds to the ID number of debris flow events (X) in the dataset, in a format like RG_FD_ID_X. All the segmented rainfall data is in “.txt” file format and stored in folders numbered by debris flow events. The “.txt” file in the folder name format is AB_CD_EF, where “AB” is the number of calculation iterations (sorting function; The smaller the “AB” value under the same directory, the closer the rainfall station is to the debris flow catchment area. “CD” is the corresponding rainfall station number, and “EF” is the date and time of the debris flow outbreak. Figure 5 shows the rainfall events and the initiation time of the debris flow. We chose to provide rainfall data for a time window starting from 7 days before and ending 1 day after the outbreak of the debris flow event. This facilitates the subsequent research on the triggering conditions of debris flow caused by early rainfall. Readers can get more detailed rainfall data from the author if needed.

### Mitigation measures of debris flow after the wenchuan earthquake

The catchment ID (C_ID), catchment name, coordinates, the number of check dams, construction time, and date of successful debris flow mitigation were included in the database (Table 3). The mitigation measures dataset is available in csv and shapefile formats in 79 watersheds after the earthquake in the study area.

**Table 3:** Structure of the mitigation measures dataset.

Filename	File type	Layers (sheets)	Attributes (columns)
DAM_DATA	Spreadsheet (.xlsx)	Dam	C_ID, catchment name, dam number, built time, successful prevention and control
D_P_DATA	Shapefile (.point)	Dam	Dam_ID, C_ID, coordinates

Attributes: C_ID: identifier of the catchment to which the mitigation measures belong; Dam_ID: identifier of the dam; built time: the completion time of the mitigation measures when putting them into use.

**Fig. 6:**
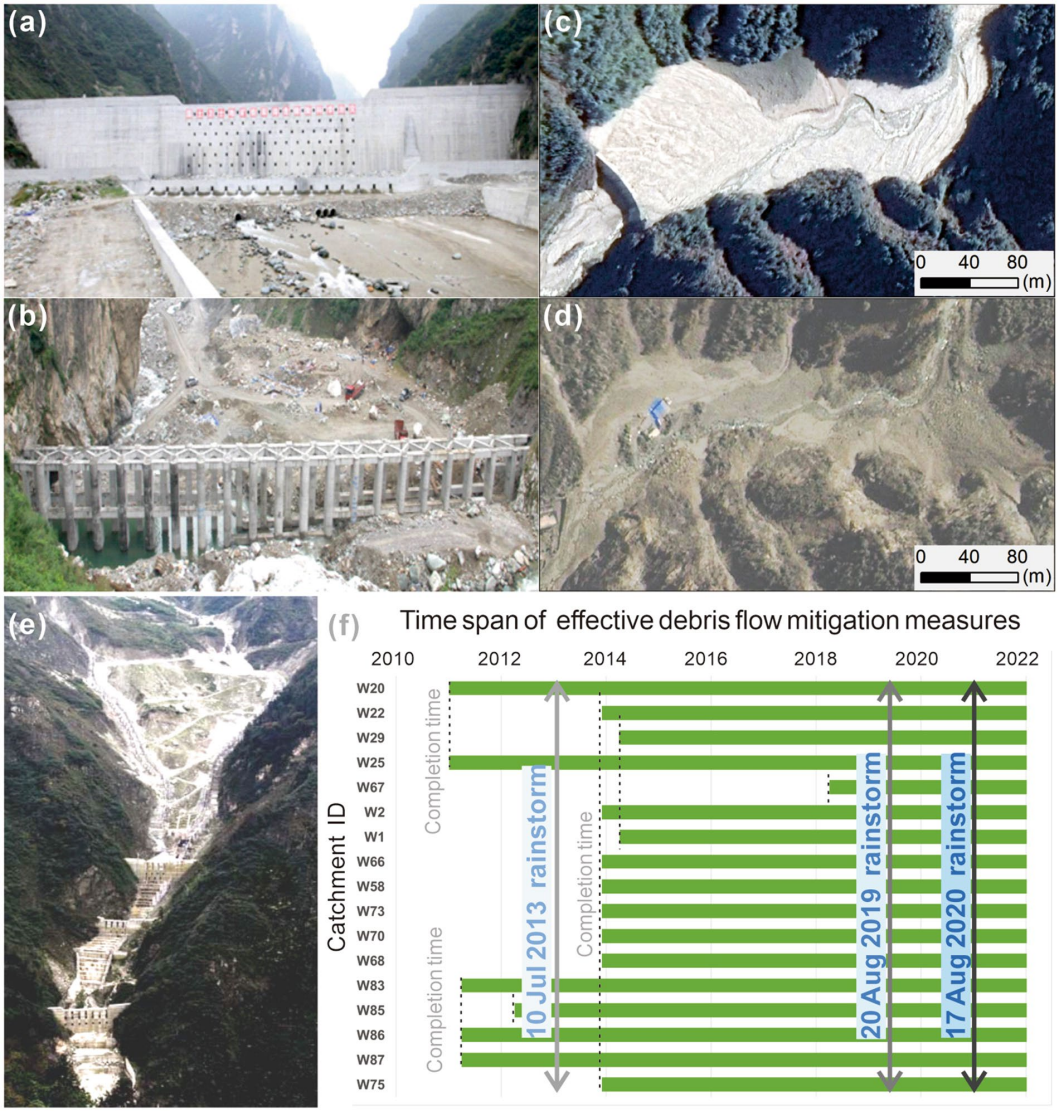
Mitigation measures, successful debris flow prevention, and control cases in the study area. (**a,b**) check dam in Qipan catchment after the 2013 debris flow; (**c**) remote sensing image after 20 August 2019 debris flow in Qipan catchment; (**d**) remote sensing image before 2013 in Qipan catchment; (**e**) UAV image in Shaofang catchment after 2010; and (**f**) the period of effective debris flow mitigation measures in the study area.

Figure 6 shows the successful case of mitigation measures and debris flow prevention and control in the study area. The results showed that effective mitigation measures were taken in 17 of the 79 watersheds in the study area during the subsequent rainstorm.

It can be seen that some mitigation measures have blocked the movement of debris flow through high-resolution remote sensing images (Fig. 6c). Figure 6(a,b) shows the mitigation measure in the Qipan catchment after the 2013 rainstorm. Figure 6(c) is the satellite remote sensing image of the retaining dam in the Qipan catchment after the 20 Aug 2019 debris flow, the image shows that the dam successfully intercepted the loose material. Figure 6(d) is the satellite remote sensing image in the Qipan catchment before the 10 Jul 2013 debris flow event. Figure 6(e) is an aerial photograph of several engineering mitigation measures constructed in the Shaofang catchment after 2010. Figure 6(f) shows the period of effective debris flow mitigation measures. After the catastrophic debris flow events in 2010 and 2013, some mitigation measures were established in the study area, which played an important role in mitigating the damage during the subsequent 2014–2020 period. These check dams are usually concrete and have extremely large stiffness^47,65^. They are usually built in densely populated areas and catchments connecting river channels. Since the debris flows move quickly and can carry boulders, the impact pressure on the inspection dam is extremely high. For example, the estimated peak impact pressure of the Wenjia catchment debris flow is about 2.4 MPa^47,49^, The loose deposits are transported to the channel gradually with time, and the back of the dam body is gradually filled with debris, which will reduce the effectiveness of the check dam^47^ (Fig. 6c,d). Therefore, it is necessary to dredge the debris behind these check dams in time before the rainy season to prevent debris flows and dam breaking.

## Technical Validation

Due to the lack of adequate monitoring equipment in the study area, the initiation time of debris flows is usually difficult to measure^41,66^. In addition to catastrophic debris flows that have been well studied and reported, there are still much small-scale debris flows in the study area that may not have been confirmed^36,41^. Therefore, the actual number of debris flows in the study area is much higher than the recorded number in our dataset. Rainfall stations are selected based on the distance between the rain gauge and the catchment, and due to differences in rainfall spatial distribution, this can make the selected data very different from the real data in debris flow initiation. The rainfall monitoring equipment of the government meteorological bureau is generally set near the ditch mouth or villages with low altitudes, but it is rarely installed near the source area. Therefore, when debris flows initiation, observed rainfall may be significantly less than actual rainfall. Due to the limitation of image resolution and time, there are errors in determining the time of disaster interpretation and mitigation measures. For example, the images we collected after 2011 were taken in December 2014 to identify debris flow and mitigation measures in 2013, but it was impossible to distinguish the debris flow and mitigation measures after 2013 and before December 2014.

## Usage Notes

We presented a multi-temporal debris flow and triggering rainfall in the Wenchuan earthquake-affected area from 2008 to 2020 and a list of debris flow mitigation measures from 2008 to 2019. The two available datasets can be used to investigate the temporal patterns of accelerated mass wasting produced by a strong earthquake and assess the effectiveness of debris flow prevention methods.

## Data Availability

There is no custom code produced during the collection and validation of this dataset.
